# Accumulation of Misfolded SOD1 in Dorsal Root Ganglion Degenerating Proprioceptive Sensory Neurons of Transgenic Mice with Amyotrophic Lateral Sclerosis

**DOI:** 10.1155/2014/852163

**Published:** 2014-04-27

**Authors:** Javier Sábado, Anna Casanovas, Olga Tarabal, Marta Hereu, Lídia Piedrafita, Jordi Calderó, Josep E. Esquerda

**Affiliations:** Unitat de Neurobiologia Cellular, Departament de Medicina Experimental, Facultat de Medicina, IRBLLEIDA, Universitat de Lleida and Institut de Recerca Biomèdica de Lleida, Avenida Rovira Roure 80, Lleida, 25198 Catalonia, Spain

## Abstract

Amyotrophic lateral sclerosis (ALS) is an adult-onset progressive neurodegenerative disease affecting upper and lower motoneurons (MNs). Although the motor phenotype is a hallmark for ALS, there is increasing evidence that systems other than the efferent MN system can be involved. Mutations of *superoxide dismutase 1* (SOD1) gene cause a proportion of familial forms of this disease. Misfolding and aggregation of mutant SOD1 exert neurotoxicity in a noncell autonomous manner, as evidenced in studies using transgenic mouse models. Here, we used the SOD1^G93A^ mouse model for ALS to detect, by means of conformational-specific anti-SOD1 antibodies, whether misfolded SOD1-mediated neurotoxicity extended to neuronal types other than MNs. We report that large dorsal root ganglion (DRG) proprioceptive neurons accumulate misfolded SOD1 and suffer a degenerative process involving the inflammatory recruitment of macrophagic cells. Degenerating sensory axons were also detected in association with activated microglial cells in the spinal cord dorsal horn of diseased animals. As large proprioceptive DRG neurons project monosynaptically to ventral horn MNs, we hypothesise that a prion-like mechanism may be responsible for the transsynaptic propagation of SOD1 misfolding from ventral horn MNs to DRG sensory neurons.

## 1. Introduction

Amyotrophic lateral sclerosis (ALS) is a devastating adult-onset neurodegenerative disease which affects upper and lower motoneurons (MNs) and causes progressive paralysis and atrophy of voluntary muscles. Death usually occurs as a result of respiratory failure, 3–5 years after the onset of clinical symptoms [1, 2]. While the majority of ALS cases are sporadic, 10% are familial (fALS), with an autosomal pattern of inheritance. A variety of mutations in the homodimeric protein Cu/Zn superoxide dismutase (SOD1) have been linked to 20% of fALS cases [3] and transgenic mice carrying mutated human SOD1 have been extensively employed as a model to investigate both familial and sporadic ALS [4, 5]. Although the motor phenotype derived from corticospinal tract and peripheral motor nerve degeneration is a hallmark of ALS, there is increasing evidence that ALS could be a multisystem disorder affecting also the somatosensory cortex [6], autonomic system [7], spinocerebellar tracts [8], and serotoninergic neurons [9]. The involvement of the peripheral sensory system has also been reported in ALS patients, particularly after electrophysiological examination [10] and also in mutant SOD1 mouse models [11]. However, the evidence of pathological changes in peripheral sensory neurons is scarce. In a previous study using SOD1 ALS murine models, we showed that an antibody which cross-reacted with neurotoxic species of mutant SOD1 provided an excellent tool for revealing this pathology in other neuronal types besides spinal cord MNs [12, 13]. In these studies we showed that ALS-linked neurodegenerative pathology could also be detected in motor cortex MNs and in other less expected CNS regions, such as serotonin-containing neurons in the raphe, noradrenergic neurons in the locus coeruleus, and Purkinje neurons in the cerebellum.

Here, we report that using our anti-misfolded SOD1 antibodies [14] it was also possible to detect degenerating sensory neurons in the dorsal root ganglion (DRG) of ALS SOD1^G93A^ mice. Degenerating sensory axons in spinal cord dorsal nerve roots were also found in parallel with the progression of the disease. Dying DRG neurons displayed a nonapoptotic phenotype and recruited macrophage cells in a similar way to that observed in ventral horn MNs. These results suggest that the fundamental mechanisms by which mutant SOD1 exerts neurotoxicity are not neuronal type-specific.

## 2. Materials and Methods

### 2.1. Animals and Tissue Preparation

The transgenic animals used in this study were B6SJL-Tg (SOD1-G93A) 1Gur/J (SOD1^G93A^) mice obtained from Jackson Laboratory (Bar Harbor, ME, USA). Once symptoms had developed, disease progression was quite rapid and caused the death of most of the animals within 128.9 ± 9.1 days. All the experimental procedures were approved by the Ethical Committee for Animal Testing of the University of Lleida in line with the norms of the Generalitat de Catalunya (DOGC 2073, 1995).

For light microscopy immunocytochemistry, the animals were deeply anaesthetized with pentobarbital, and transcardially perfused with physiological saline solution followed by 4% paraformaldehyde (PFA) in 0.1M phosphate buffer (PB) at pH 7.4. After 24 hours in PFA, samples were transferred to 30% sucrose in 0.1 M PB and 0.02% sodium azide for cryoprotection and were then frozen for cryostat sectioning.

For electron microscopic examination, animals were perfused with 1% PFA and 1% glutaraldehyde in 0.1 M PB at pH 7.4. DRG and their ventral and dorsal nerve roots (VRs and DRs, resp.) were separately dissected and processed: they were then postfixed in 1% osmium tetroxide and embedded with Embed 812 epoxy resin according to standard procedures. Ultrathin sections were counterstained with uranyl acetate and lead citrate and observed in a Zeiss EM 910 (Zeiss, Oberkochen, Germany) electron microscope. Semithin sections (1 *μ*m thick) stained with methylene blue were also examined and imaged using an Olympus 60X/1.4 NA PlanApo oil immersion objective (Olympus, Hamburg, Germany) and a DMX 1200 Nikon (Tokyo, Japan) digital camera.

### 2.2. Immunohistochemistry and Image Analysis

Cryostat sections (16 *μ*m thick) of PFA-fixed DRGs were pretreated with phosphate buffered saline (PBS) containing 0.1% Triton X-100, blocked in 3% normal goat serum, and incubated overnight with primary antibodies at 4°C. The primary antibodies used were the following: (1) AJ10 antibody was produced in our laboratory by immunizing rabbits with a human SOD1 peptide sequence (VKVWGSIKGLTEGLHGFHVHEFGDNTAGC); this antibody is specific for misfolded ALS-linked mutant human SOD1conformers and barely reacts with wild-type (WT) human SOD1 [14] rabbit polyclonal anti-P2X4 (1 : 500; Alomone Labs, Jerusalem, Israel); some lots of this antibody, such as lot AN-04 and AN-05, cross-react with high affinity with ALS-linked misfolded conformers of SOD1 [12, 13]; (3) rat monoclonal anti-Mac-2 (1 : 800, Antibodies online GMBH, Aachen, Germany); (4) rat monoclonal anti-mouse CD68 (1 : 100; AbD Serotec, Oxford, UK); (5) mouse monoclonal anti-calcitonin gene-related peptide (CGRP, 1 : 100; Abcam, Cambridge, UK; ab81887); (6) goat polyclonal anti-parvalbumin (PV, 1 : 1000; Swant, Marly, Switzerland; pv-235); (7) guinea pig polyclonal anti-substance P (SP, 1 : 1000; Abcam; ab10353); and (8) mouse monoclonal anti-neuron-specific class III *β*-tubulin (TUJ1, 1 : 500, RD Systems, Minneapolis, MN, USA; mab1195).

After washing in PBS, sections were incubated with appropriate secondary antibodies (1 : 500) which were labeled with either Alexa Fluor 488 (Invitrogen), Cy3, or Cy5 (Jackson Immunoresearch Laboratories, West Grove, PA, USA). Sections were treated with 4′,6-diamidino-2-phenylindole dihydrochloride (DAPI, 50 ng/mL) for nuclear fluorescent staining. Some sections were also incubated with the isolectin B4 from* Bandeiraea simplicifolia* conjugated with fluorescein isothiocyanate (FITC, 1 mg/mL, Sigma).

Mounted slices were examined and imaged with an Olympus BX51 epifluorescence microscope equipped with a DP30BW camera or a FluoView 500 Olympus confocal laser-scanning microscope.

Morphometry was performed on digital images using ImageJ (National Institutes of Health, Bethesda, MA, USA) or Visilog 6.3 software (Noesis, Orsay, France).

### 2.3. Statistical Analysis

All data are expressed as mean ± SEM. The statistical analysis was assessed using either Student's *t*-test or one-way analysis of variance (ANOVA) followed by a post hoc Bonferroni's test. The level of significance was chosen as *P* < 0.05.

## 3. Results

### 3.1. Nerve Pathology in Ventral and Dorsal Roots of SOD1^G93A^ Mice

**Figure 1:**
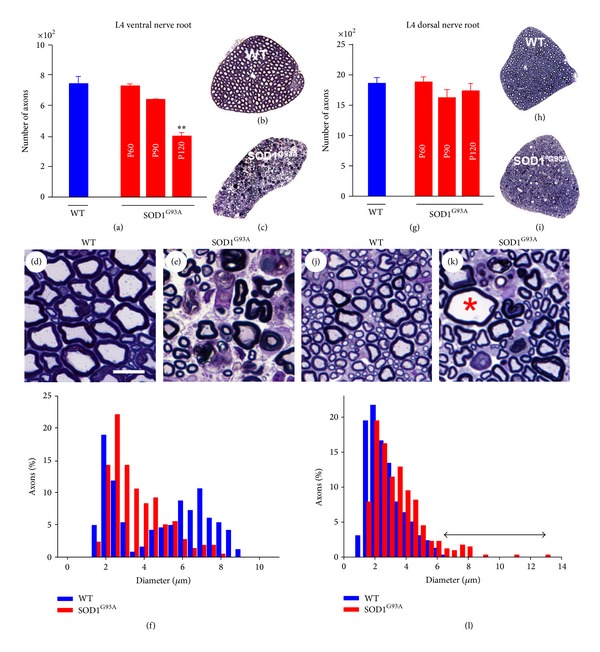
Morphometric analysis of motor (L4 VRs, (a)–(f)) and sensory (L4 DRs, (g)–(l)) axons from WT and SOD1^G93A^ mice. An analysis was performed on 1 *μ*m semithin plastic transversal sections ((b), (c), (h), and (i)). ((a)–(c) and (g)–(i)) Counts showed a significant loss of the total number of motor, but not sensory, axons in the end-stage (P120) SOD1^G93A^ mice ((a) and (g)). ((d)–(f) and (j)–(l)) A frequency distribution analysis of myelinated axon size in WT VRs showed a clear bimodal profile (f) indicative of axons coming from *α*-MNs (large) and *γ*-MNs (small); note the selective loss of large axons in SOD1^G93A^ mice. Representative images of WT and SOD1^G93A^ ventral nerve are shown in (d) and (e); note the presence of abundant degenerating axons in SOD1^G93A^ animals. Although there was no evidence of the loss of sensory axons in dorsal nerve roots in SOD1^G93A^ mice, a more detailed examination of nerve profile morphology ((j) and (k)) revealed the presence of moderate numbers of axons exhibiting significant degrees of swelling (∗) and other degenerating features. A frequency distribution analysis of myelinated axon size in dorsal nerve roots (l) reflected the appearance of a new population of large diameter (degenerating) sensory axons in SOD1^G93A^ samples (indicated by a double arrowed line). The bars in the graphs represent the mean ± SEM values of counts performed in 2–11 animals per age and experimental condition; **P < 0.01 versus WT, one-way ANOVA, Bonferroni's post hoc test). Scale bar in *D* = 10 *μ*m (valid for (e), (j), and (k)).

The extent of sensory system involvement in ALS and its correlation with the more genuine pathology seen in motor system were analyzed. The total number of apparently healthy axons contained in L4 VRs (motor) and DRs (sensory) was counted in semithin plastic sections taken from SOD1^G93A^ mice. These animals develop overt neuromuscular clinical deficits starting from around postnatal day (P) 90; this was followed by paralysis and then death at around P130 [15, 16]. As can be observed in Figures 1(a)–1(c), the number of VR motor axons started to decline from P90 and, as expected, about 50 % of these axons had been lost by P120. A similar profile of nerve degeneration, though on a different scale, was observed for the sensory axons of the DRs. This severe nerve pathology was not, however, reflected as an axonal loss in our DR counts. This is because the degenerating sensory axons had still not disappeared in end-stage animals at the moment of sampling (Figures 1(g)–1(i)). In both the VRs and DRs, we observed more or less extensive Wallerian-like degenerative changes, respectively, with abundant myelin debris and myelin ovoids engulfed by phagocytic cells (Figures 1(d), 1(e), 1(j), and 1(k)). Whereas axonal loss mainly involved large motor axons (>6 *μ*m diameter) in VRs, in DRs, axonal depletion resulted in an unaltered frequency distribution of axonal caliber; the only exception to this was the appearance of a new population of large (>8 *μ*m diameter) swollen and abnormal axons (Figures 1(f) and 1(l)).

**Figure 2:**
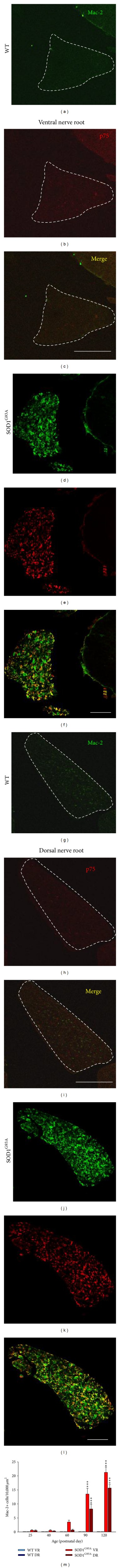
Degenerative changes in motor and dorsal axons were highlighted using Mac-2 immunolabeling (green), as a marker for activate macrophage infiltration, and p75 (a low affinity neurotrophin receptor that is upregulated in denervated Schwann cells) immunofluorescence (red). These two proteins were barely visible in the VRs ((a)–(c)) and DRs ((g)–(i)) (delimited by dashed lines) from WT animals. In contrast, Mac-2 and p75 were markedly upregulated in both the VRs ((d)–(f)) and DRs ((j)–(l)). The density of Mac-2 positive cells was quantified at different postnatal ages (m); the bars in the graph represent the mean ± SEM values for counts performed in 5 nerve roots (L4) of 3 animals per age and experimental condition; ***P < 0.001 versus its respective WT nerve root (one-way ANOVA, Bonferroni's post-hoc test). VR: ventral nerve root, DR: dorsal nerve root. Scale bars: 150 *μ*m in (c) and (i) (valid for (a), (b), (g), and (h)), 50 *μ*m in (f), and (g) (valid for (d), (e), (j), and (k)).

The density of activated phagocytic cells was evaluated using Mac-2 immunostaining. It is known that both blood-borne macrophages and Schwann cells phagocyting degenerating myelin after peripheral nerve injury display Mac-2 immunoreactivity [17]. In VRs and DRs of WT mice Mac-2 positive cells were scarce (Figures 2(a) and 2(g)). In SOD1^G93A^ animals, the density of Mac-2 positive phagocytes was found to be slightly greater in VRs at P25 and P40 (Figure 2(m)). It was, however, possible to observe a progressive increase in VR phagocytes after P60 (Figures 2(d) and 2(m)). DR phagocytes followed a similar, although damped, profile (Figures 2(j) and 2(m)).

To additionally evaluate DR pathology in SOD1^G93A^ animals, the extent of Schwann cell denervation was analyzed by detecting the expression of the low affinity nerve growth factor (NGF) receptor, p75. This receptor has been found to be dramatically upregulated in Schwann cells after peripheral nerve injury [18, 19]. Whereas no p75 positive cells were found in VRs and DRs from WT animals (Figures 2(b), 2(c), 2(h), and 2(i)), p75 was found highly to be upregulated in both nerve roots of SOD1^G93A^ mice during the end-stage of the disease (Figures 2(e), 2(f), 2(k), and 2(l)), with the reaction being stronger in VRs (the numbers of p75 positive cells/1,000 *μ*m^2^ were 2.3 ± 0.1 for VRs and 1.5 ± 0.1 for DRs; *n* = 30 fields, 3 mice per condition, *P* < 0.001, Student's *t*-test).

**Figure 3:**
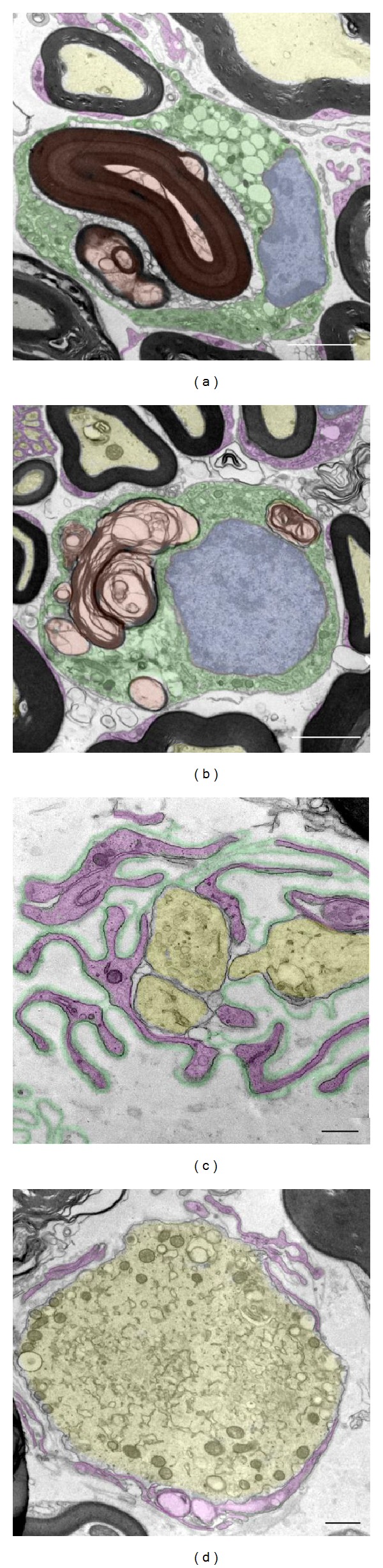
The ultrastructural morphology of VRs ((a), (c), and (d)) and DRs (b) from end-stage SOD1^G93A^ mice. From a qualitative point of view, similar Wallerian-like degenerative changes could be observed in both VRs (a) and DRs (b); phagocytic cells (shaded in green), in which the nucleus is dashed in blue, appear to engulf the myelin debris (shaded in red); apparently normal myelinated axons are dashed in yellow and Schwann cell profiles are dashed in magenta. ((c)–(d)) In ventral roots it is possible to observe some regenerating axons (yellow in (c)) growing inside the ghosts of folded basal lamina profiles (green in (c)) filled with Schwann cell pedicles (magenta in (c)). Occasionally, a large growth cone (yellow in (d)) filled with organelles and surrounded by Schwann cell profiles (magenta in (d)) can be detected. Scale bars: 5 *μ*m in (a), 2.5 *μ*m in (b), 0.5 *μ*m in (c), and 1 *μ*m in (d).

After electron microscopy examination, it was found that both VRs and DRs underwent similar qualitative degenerative changes. These changes were highly comparable to those described during Wallerian degeneration, which takes place in the nerve segment distal to the site of lesion after axonal transection [20]. The most conspicuous alteration was extensive myelin degradation, with the formation of myelin ovoids and the presence of phagocytic cells engulfing large amounts of lamellar myelin debris (Figures 3(a) and 3(b)). Signs of axonopathy, such as axonal swelling, abnormal accumulations of organelles, and altered mitochondria, were also seen. Ultrastructural signs of axonal regeneration were also observed, but only in VRs; these involved the presence of folded basal lamina sheaths in which some Schwann cell processes enveloped thin growing axon profiles (Figures 3(c) and 3(d)). The folded basal lamina tubes represented empty “ghosts” of the original nerve fibers that were lost after degeneration and served as a scaffold for newly formed axonal sprouts (Figure 3(c)). Giant axonal profiles filled by vesicular organelles, mitochondria, and cytoskeletal filaments surrounded by thin nonmyelinating Schwann cells were also observed (Figure 3(d)). This organization was typical of the growth cone ultrastructure. These results suggest that although analogous degenerative pathomorphological changes were present in sensory and motor axons, the regenerative response was only detected in the latter.

### 3.2. Dorsal Root Ganglion Cell Degeneration in SOD1^G93A^ Mice

**Figure 4:**
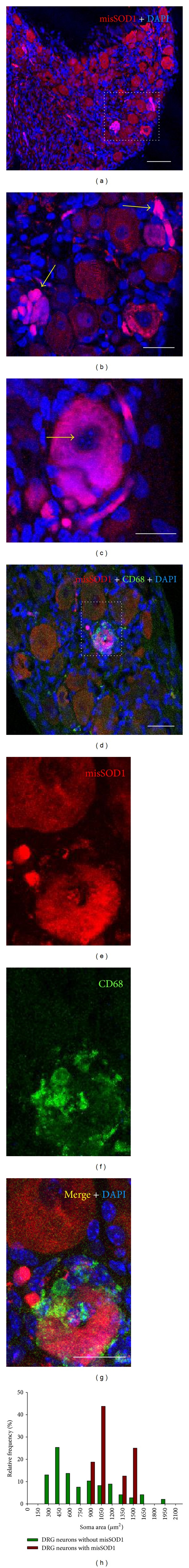
((a)–(c)) Misfolded SOD1 (misSOD1) immunolabeling using AJ10 antibody (red) reveals highly immunoreactive neuronal somata within the DRG (delimited in (a)) of end-stage (P120) SOD1^G93A^ mice. Some neuronal somata display cytoplasmic fragmentation indicative of degenerative changes (arrows in (b)); nuclei, counterstained with DAPI (blue), do not show apoptotic morphology (arrows in (c)). ((d)–(g)) Misfolded SOD1 immunolabeling (red) was combined with an anti-CD68 antibody (green) to reveal activated macrophagic cells; DAPI (blue) was used for nuclear staining. A degenerating neuron expressing misfolded SOD1 (delimited in (d)) and displaying clustered profiles of CD68 positive phagocytic cells is shown in high magnification in (e)–(g). (h) A frequency distribution profile of the size of the DRG neuron somata containing misfolded SOD1 with respect to that of the whole neuronal population; note that misfolded SOD1 positive neurons belong to the large-sized (presumably proprioceptive) population. Scale bars: 80 *μ*m in (a), 40 *μ*m in (b), (c), (d), and (g) (also valid for (e) and (f)).

Misfolded SOD1 accumulation in MN cell bodies has been shown to be useful tool for monitoring cellular dysfunction in SOD1 mouse models of ALS [21]. We generated an antibody which was able to recognize misfolded conformations of SOD1 shared by different ALS-linked mutations [14]. Here, this antibody was used to explore whether sensory axon degeneration involved the accumulation of misfolded SOD1 in DRG neuronal cell bodies. In end-stage animals, some DRG neurons displayed strong misfolded SOD1 immunoreactivity which sometimes extended to neuritic profiles (Figures 4(a)–4(c)). Some of these presented signs of cytoplasmic fragmentation indicative of cell body disruption. As already described in MN cell bodies, degenerating DRG neurons with misfolded SOD1 recruited phagocytic cells; this was demonstrated using the macrophage/microglia marker CD68 (Figures 4(d)–4(g)). The neurons with misfolded SOD1 accumulation were significantly larger than the rest of the DRG neuronal population (mean area in *μ*m^2^, misSOD1^+^ neurons: 1099.6 ± 52.5, *n* = 16, and misSOD1^−^ neurons: 706.2 ± 34.3, *n* = 146, *P* < 0.001; mean diameter in µm, misSOD1^+^ neurons: 36.9 ± 1, *n* = 16, and misSOD1^−^ neurons: 28.7 ± 0.7, *n* = 146, *P* < 0.001; Student's *t* test). When individual values were plotted in a histogram frequency graph, the selective involvement of the large cell population in misfolded SOD1 accumulation was clearly seen (Figure 4(h)).

**Figure 5:**
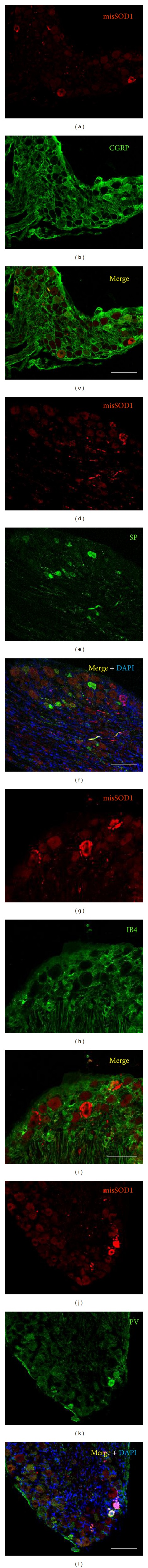
Micrographs taken from the DRGs of end-stage (P120) SOD1^G93A^ mice. Misfolded SOD1 (misSOD1) immunolabeling using AJ10 antibody (red) was combined with several markers for DRG neuronal types, as indicated (green). ((a)–(i)) Misfolded SOD1 immunoreactive neurons do not colocalize with the neuropeptides CGRP and SP, or with IB4, all of which are markers for neuronal types other than proprioceptive. ((j)–(l)) However, neurons containing misfolded SOD1 colocalize with PV, a marker for proprioceptive sensory neurons. In some cases, DAPI was used for nuclear staining (blue). The prominent background observed in (c) and (d) is due to an unspecific connective tissue reaction involving the secondary antibody when a mouse monoclonal was used as a primary antibody. Scale bars = 200 *μ*m.

It is possible to further distinguish the heterogeneous cellular population present in DRG according to specific cytochemical properties. CGRP and SP are contained in small, unmyelinated, peptidergic, and nociceptive primary sensory neurons; the isolectin IB4 is also a marker for small, unmyelinated, but nonpeptidergic, sensory neurons; and PV labels large, proprioceptive, neurons that innervate muscle spindles [22, 23]. All of these markers were used here in order to determine whether misfolded SOD1 had accumulated in a particular subpopulation of DRG neurons. We did not observe any misfolded SOD1 positive neurons containing CGRP, SP, or IB4 labeling (Figures 5(a)–5(i)). However, the misfolded SOD1 positive neurons displayed highly intense PV immunolabeling, indicating that they belong to the proprioceptive population (Figures 5(j)–5(l)).

**Figure 6:**
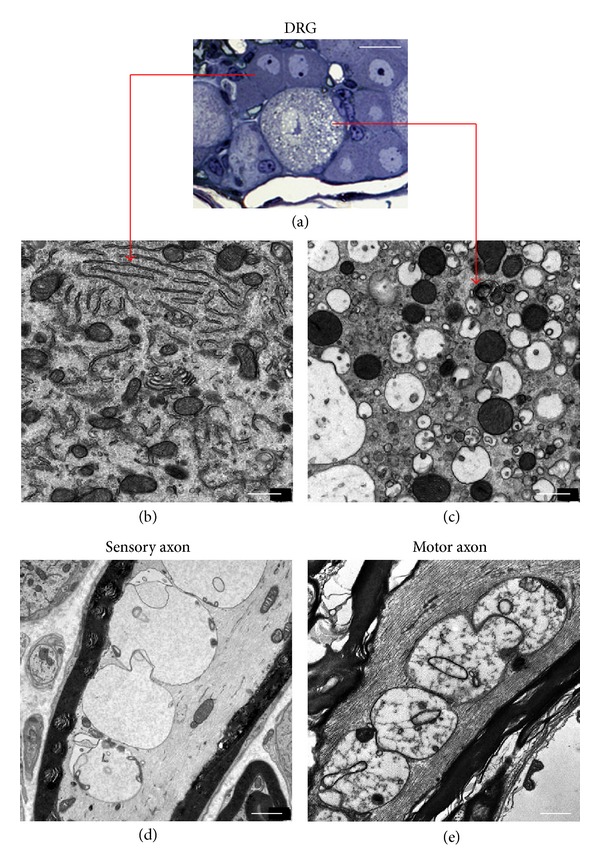
(a) Semithin plastic section of a DRG from end-stage (P120) SOD1^G93A^ mice showing a degenerating, microvacuolated, large neuronal soma, which is surrounded by apparently normal medium-sized neuronal cells. ((b) and (c)) The cells indicated in the semithin sections were examined by electron microscopy which revealed a well-preserved organelle structure in the medium-sized neuronal cell (b) and extensive microvacuolization in the degenerating large soma (c). (d) A large myelinated axonal profile adjacent to the DRG sensory neurons shows an accumulation of highly vacuolated mitochondria with a comparable morphology to that of degenerating mitochondria typically described in motor axons of SOD1^G93A^ mice (as shown in (e), taken from a sample of facial nucleus). Scale bars: 20 *μ*m in (a), 0.5 *μ*m in (b) and (c), and 1.5 *μ*m in (d) and (e).

Large neurons with degenerative changes can also be easily detected in semithin sections of DRGs from terminal SOD1^G93A^ mice. Microvacuolization was the main alteration observed in these neurons. Their nuclei did not display the typical apoptotic morphology but appeared shrunken and exhibited a loss of their normal, well-defined circular shape (Figure 6(a)). Under electron microscopy, the vacuoles displayed a round profile and were delimited by a membrane, suggesting that they originate from the vacuolar disruption of the endoplasmic reticulum. The mitochondria showed a round, condensed morphology (Figure 6(c), compared with Figure 6(b)). Large myelinated axons adjacent to degenerating DRG neuronal cell bodies often exhibited an accumulation of highly prominent vacuolated mitochondria which displayed the typical alteration that has been described in motor axons and dendrites of SOD1^G93A^ mice (Figures 6(d) and 6(e)).

**Figure 7:**
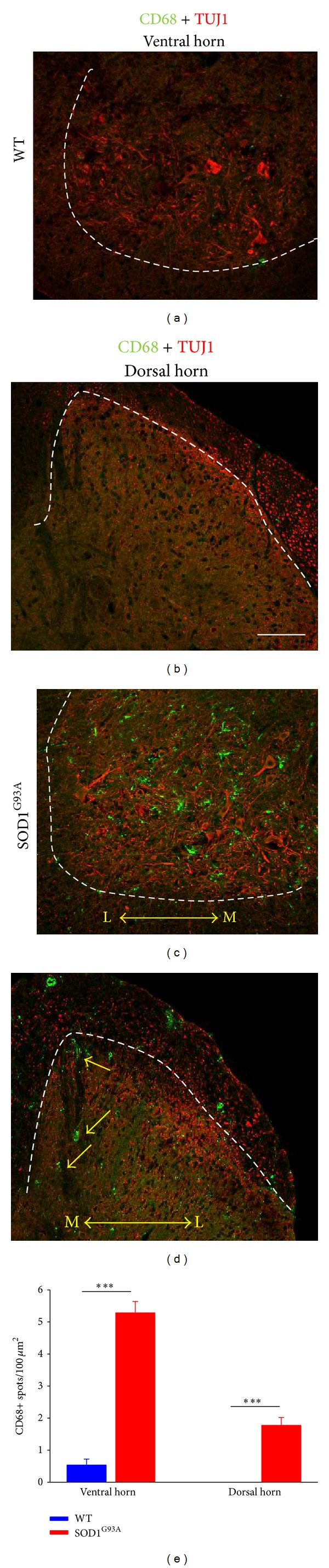
((a)–(d)) Double immunolabeling with TUJ1 (to label neuronal profiles, red), and anti-CD68 (to reveal microglial cells, green) antibodies in ventral ((a), (c)) and dorsal ((b), (d)) horn of WT ((a), (b)) and end-stage SOD1G93A ((c), (d)) mice; L: lateral, M: medial. CD68-positive profiles were rarely detectable in WT samples, but they were present in either large or moderate quantities in the ventral and dorsal horn, respectively, of SOD1^G93A^ samples. In SOD1^G93A^ dorsal horns, it was possible to observe some infiltrating microglial cells adjacent to medially located (presumably proprioceptive) axon fascicles, entering the spinal cord (arrows in (d)). (e) Quantification of CD68-positive profiles in the spinal cord of WT and end-stage SOD1^G93A^ mice. Bars represent mean ± SEM values for counts performed in 6–11 fields of 2 animals per experimental condition; ***P < 0.001 versus WT (one-way ANOVA, Bonferroni's post hoc test). Scale bar in (b) = 100 *μ*m (also valid for (a), (c), and (d)).

To assess whether degenerating sensory axons entering the spinal cord involves the activation of microglial cells, the density of CD68-positive microglial profiles was analyzed in dorsal areas of spinal cord gray matter (Figures 7(a)–7(e)). For comparisons, counts were also performed in ventral horn gray matter, in which degenerating MNs were located and neuroinflammatory microglial activation has already been well established in the model. As expected, we found a notable infiltration of CD68 positive microglia around motoneuronal somata. A moderate increase in CD68 positive microglial cells was also observed in the dorsal gray matter; some of these profiles were seen adjacent to medially located fascicles of sensory axons corresponding to sensory axons originating from proprioceptive sensory neurons [24].

## 4. Discussion

Although ALS is mainly characterized by motor axon degeneration and the loss of lower and upper MNs, there is a considerable amount of data concerning the abnormalities in the sensory system in both human and experimental models. For instance, neuropathological studies have demonstrated reductions of more than 50% in large L5 DRG cell bodies [25], while substantial degeneration of sensory axons in the sural and peroneal nerves has also been observed in ALS patients [26–28]. By means of neurophysiological explorations, it has been shown that 20–60% of ALS patients have abnormalities in their sensory system [10, 29]. Consistent with human pathology, sensory nerve degeneration has also been evidenced in SOD1^G93A^ mouse models [11, 30, 31]. Here, we confirm that DRG sensory neurons and their axons are clearly damaged in ALS SOD1^G93A^ mice and show that misfolded SOD1 accumulation is on the base of this process. Misfolded SOD1 accumulation in DRG neurons also elicits a neuroinflammatory response which is comparable to that observed in ventral horn MNs [13, 14]; this indicates that the same basic pathophysiological process, which leads to neuronal loss, is involved in both cell populations. Degenerating DRG neuronal somata displayed an extensive vacuolar pathology, probably originated from disrupted ER, in the absence of apoptotic nuclear changes. Myelinated axonal profiles adjacent to DRG neuron somata often showed extremely vacuolated mitochondria, with identical morphologies to those characteristically observed in motor axons [32–34]. As these alterations appear to be induced by aggregates of misfolded SOD1 in mitochondria and to correlate with the exacerbation of disease [35, 36], their presence within sensory axons would seem to point to a common SOD1-dependent mechanism of mitochondrial swelling shared by both motor and sensory axons.

From a qualitative point of view, degenerating changes in the VRs and DRs displayed a Wallerian-like morphology, with the alterations being substantially exacerbated in the VRs. One of the early changes in sectioned axons is the dissolution of axoplasm, with the subsequent loss of cytoskeletal components, organelles, and axolemma. This results in a swollen axoplasm surrounded by intact myelin [20], which is the predominant morphology we observed in the DRs. Later, myelin debris appears engulfed by phagocytic/Schwann cells leading to axonal loss when regenerative events are unable to compensate for the injury. This is the morphological pattern usually seen in VRs. However, this reduction in axonal numbers is only prominent in VRs because the progression of the degenerative process leading to axonal loss in DRs is halted as a result of animal death. It should also be taken into account that since there is no clear cut distinction between normal and early degenerating axons in semithin sections, the latter were also included in our counts.

Vacuolar degeneration is a common feature of the DRG neuronal degeneration observed in a variety of conditions including diabetes [37], Charcot-Marie-Tooth disease [38], and bortezomib-induced neurotoxicity [39]. In our model, extensive microvacuolization was observed in DRG neurons that had presumably accumulated misfolded SOD1. Agents like bortezomib are potent endoplasmic reticulum (ER) stressors and lead to its vacuolar disruption. As misfolded SOD1 has been shown to play a major role in ALS MN degeneration by inducing ER stress [40, 41], the microvacuolization that we found in DRG neurons should be understood as a consequence of misfolded SOD1-induced ER stress in a type of neuron which is not usually considered a main target in ALS.

One question which arises from our results is whether misfolded-SOD1 accumulation in DRG neurons is a primary cell process similar to that occurring in ventral horn motoneurons or, on the other hand, a consequence of cell-to-cell spreading of misfolded-SOD1 conformers via a prion-like mechanism. We should point out that the DRG neuronal subpopulation that degenerate as a result of misfolded-SOD1 accumulation are large proprioceptive neurons, some of which establish monosynaptic contacts with ventral horn MNs. As it has been demonstrated* in vitro* that SOD1 misfolding and aggregation can propagate in a prion-like manner in neuronal cells [42, 43] and it has been strongly suggested that this mechanism could also operate* in vivo* [44], our findings may provide support for this hypotheses.

## 5. Conclusion

The misfolded SOD1-dependent degeneration of proprioceptive neurons that we have described here is a late event in the natural history of ALS in the SOD1^G93A^ fast mouse model and could be consistent with a prion-like spreading mechanism that emerges in advanced stages of the disease. A more precise determination of the alteration in the proprioceptive system involving noninvasive procedures could be useful as a biomarker of the progression of disease.

## Abbreviations

ALS: Amyotrophic lateral sclerosis (ALS)
CGRP: Calcitonin gene-related peptide
CNS: Central nervous system
DAPI: 4′,6-Diamidino-2-phenylindole dihydrochloride
DR: Dorsal nerve root
DRG: Dorsal root ganglion
ER: Endoplasmic reticulum
fALS: Familial form of amyotrophic lateral sclerosis
FITC: Fluorescein isothiocyanate
IB4: *Bandeiraea simplicifolia* isolectin B4
misSOD1: Misfolded SOD1
MN: Motoneuron
P: Postnatal day
PB: Phosphate buffer
PFA: Paraformaldehyde
PV: Parvalbumin
SOD1: Superoxide dismutase 1
SP: Substance P
VR: Ventral nerve root.
